# Aldehyde dehydrogenase, Ald4p, is a major component of mitochondrial fluorescent inclusion bodies in the yeast *Saccharomyces cerevisiae*

**DOI:** 10.1242/bio.20147138

**Published:** 2014-04-25

**Authors:** Yoshiko Misonou, Maiko Kikuchi, Hiroshi Sato, Tomomi Inai, Tsuneyoshi Kuroiwa, Kenji Tanaka, Isamu Miyakawa

**Affiliations:** 1 Department of Biology, Faculty of Science, Yamaguchi University, Yamaguchi 753-8512, Japan; 2 Department of Life Science, College of Science, Rikkyo University, Tokyo 171-8501, Japan; 3 Core Research for Evolutional Science and Technology (CREST), Japan Science and Technology Agency, Gobancho, Chiyoda-ku, Tokyo 102-0076, Japan; 4 Laboratory of Medical Mycology, Research Institute for Disease Mechanism and Control, Nagoya University School of Medicine, Nagoya 466-8550, Japan; * Present address: Division of Cell Biology, Institute of Life Science, Kurume University, Hyakunen-kohen 1-1, Kurume, Fukuoka 839-0864, Japan.; ‡ Present address: Department of Microbiology, Aichi Gakuin University School of Dentistry, Kusumoto-cho, Chikusa-ku, Nagoya 464-8650, Japan.

**Keywords:** Yeast, Mitochondria, Inclusion body, Aldehyde dehydrogenase, Ald4p

## Abstract

When *Saccharomyces cerevisiae* strain 3626 was cultured to the stationary phase in a medium that contained glucose, needle-like structures that emitted autofluorescence were observed in almost all cells by fluorescence microscopy under UV excitation. The needle-like structures completely overlapped with the profile of straight elongated mitochondria. Therefore, these structures were designated as mitochondrial fluorescent inclusion bodies (MFIBs). The MFIB-enriched mitochondrial fractions were successfully isolated and 2D-gel electrophoresis revealed that a protein of 54 kDa was only highly concentrated in the fractions. Determination of the N-terminal amino acid sequence of the 54-kDa protein identified it as a mitochondrial aldehyde dehydrogenase, Ald4p. Immunofluorescence microscopy showed that anti-Ald4p antibody specifically stained MFIBs. Freeze-substitution electron microscopy demonstrated that cells that retained MFIBs had electron-dense filamentous structures with a diameter of 10 nm in straight elongated mitochondria. Immunoelectron microscopy showed that Ald4p was localized to the electron-dense filamentous structures in mitochondria. These results together showed that a major component of MFIBs is Ald4p. In addition, we demonstrate that MFIBs are common features that appear in mitochondria of many species of yeast.

## INTRODUCTION

Yeast mitochondria dynamically change their morphology depending on the life cycle stage of cells, as well as on changes in the metabolic state of cells. Changes in the number and morphology of mitochondria in *S. cerevisiae* have been extensively studied by electron microscopy of both vegetative and sporulating cells (Stevens, 1981). On the other hand, fluorescence microscopy provides us with a convenient means to observe the morphology of mitochondria or mitochondrial nucleoids in a population of cells with or without chemical fixation. To date, we have studied changes in the morphology of mitochondria and mitochondrial nucleoids of *S. cerevisiae* during the life cycle. We have found that many oval mitochondria, up to 60 in a stationary-phase cell, begin to fuse together to form a tubular mitochondrial network during meiotic prophase, as shown by 4′,6-diamidino-2-phenylindole (DAPI) staining of fixed cells and by vital staining of cells with dimethyl aminostyrylmethylpyridiniumiodine (DASPMI). Thereafter, the tubular mitochondrial network is divided and distributed into 4 spores in a characteristic manner (Sando et al., 1981; Miyakawa et al., 1984). Morphological changes of mitochondria during meiosis and the sporulation process can be vitally visualized by double-staining of cells with DAPI and 3,3′-dihexyloxacarbocyanine iodide {DiOC_6_(3)} (Miyakawa et al., 1994).

Mitochondria also have a highly tubular structure in growing cells at log-phase, and they migrate into the buds with one end of the tubular mitochondria leading the way, as shown by DAPI and DiOC_6_(3) staining or by labeling of mitochondria with GFP (Miyakawa et al., 2010). Migration of tubular mitochondria into the bud cells has also been clearly shown in the triangular yeast *Trigonopsis variabilis* by vital staining with DiOC_6_(3) (Miyakawa and Yanagamizu, 1998). Similarly, formation of a tubular network of fused mitochondrion has been shown by DiOC_6_(3) staining of *Saccharomycodes ludwigii* during meiosis and sporulation (Miyakawa et al., 2012).

During the course of vital staining of mitochondria in various strains of *S. cerevisiae* and other species of yeast, we frequently found straight needle-like structures in stationary-phase cells without chemical fixation that emitted blue-white autofluorescence under UV excitation. The frequency of appearance of fluorescent needle-like structures largely depended on the strains used. Interestingly, these structures always overlapped with outlines of straight elongated mitochondria stained with DiOC_6_(3). Accordingly, we tentatively designated these structures as mitochondrial fluorescence inclusion bodies (MFIBs). Among the observed *S. cerevisiae* strains, MFIBs were most frequently observed in more than 80% of stationary-phase cells of a particular strain. Autofluorescence of MFIBs is available to identify MFIB-enriched mitochondrial fractions during fractionation of cell homogenate. Then, we considered that biochemical analysis of MFIBs would be possible by isolating MFIB-containing mitochondria.

In the last three decades, three electron microscopic observations on the intramitochondrial fibrous inclusion bodies of yeasts have been reported (May, 1974; Nagano et al., 1982; Yotsuyanagi, 1988). May found bundles of filaments with a diameter of 5 nm in elongated mitochondria in the yeast *Saccharomyces carlsbergensis* fixed with glutaraldehyde and osmium (May, 1974). It was suggested that actomyosin-like contractile proteins were present in mitochondria from the structural similarity. Nagano et al. also reported the presence of fibrous structures in the mitochondrial matrix in cells of *Kloeckera *sp. that were grown in a culture medium containing methanol and in isolated mitochondria by the freeze-etching method combined with a rapid freezing procedure (Nagano et al., 1982). Each filament exhibited a helical structure with periodic lengths of 10 and 13 nm. They assumed that the fibrous structures consisted of DNA. Yotsuyanagi found similar fibrous structures, designated intramitochondrial fiber (IMF), in the electron microscopic observation of yeasts, *Saccharomyces cerevisiae* and *Saccharomyces uvarum* (Yotsuyanagi, 1988). However, no report has presented any evidence on the chemical composition of the yeast mitochondrial inclusions.

In this study, we present several lines of evidence that the major component of MFIBs is a mitochondrial aldehyde dehydrogenase, Ald4p. This is the first biochemical characterization of yeast intramitochondrial inclusion bodies.

## RESULTS

### Appearance of fluorescent inclusion bodies in elongated mitochondria

**Fig. 1.**
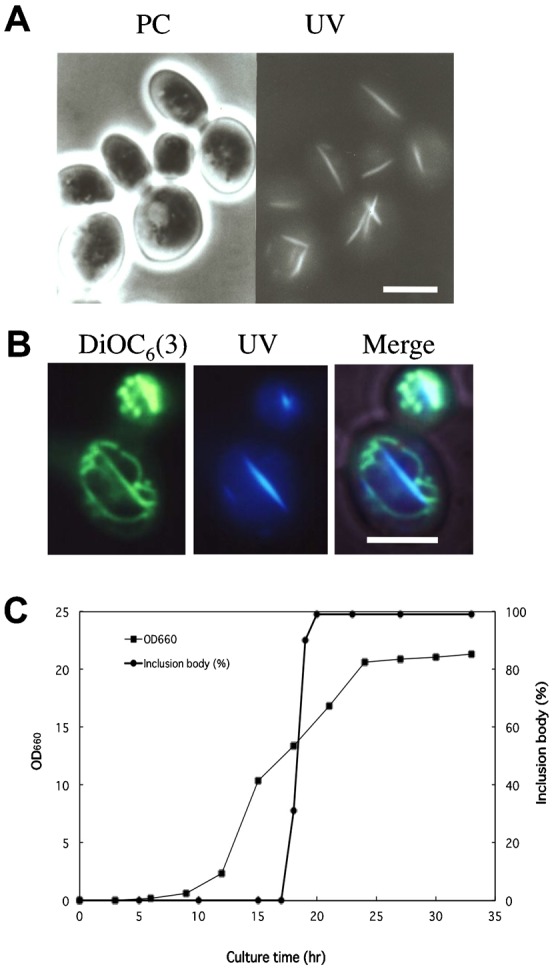
Appearance of mitochondrial fluorescent inclusion bodies (MFIBs) in *S. cerevisiae*. (A) Cells of the strain 3626 were cultured to the stationary phase in YPD medium and observed by phase contrast microscopy (PC) and fluorescence microscopy under UV excitation (UV). (B) Cells were vitally stained with DiOC_6_(3) and observed by fluorescence microscopy under B excitation for mitochondria, under UV excitation for MFIBs, and the two images were merged. (C) Changes in percentage of cells that retained MFIBs during liquid culture. *S. cerevisiae* strain 3626 was cultured in YPD medium. (▪) Optical density at 660 nm. (•) Percentage of cells that retained MFIBs. Scale bars: 5 µm.

When the 3626 strain of *S. cerevisiae* that was cultured to the stationary phase was observed under UV excitation by fluorescence microscopy, needle-like structures with an average length of 4.6 µm that emitted autofluorescence were observed in almost all cells (Fig. 1A). The fluorescent inclusion bodies could be observed only in living cells, and the autofluorescence rapidly disappeared by fixation of cells with glutaraldehyde, formaldehyde, or ethanol. When living cells were stained with DiOC_6_(3) to observe the mitochondria, the fluorescent inclusion bodies completely overlapped with the contour of the elongated mitochondria (Fig. 1B). A number of observations showed that the fluorescent inclusion bodies strictly coincided with the elongated mitochondria in cells. Therefore, we referred to these structures as mitochondrial fluorescent inclusion bodies (MFIBs). As shown in Fig. 1A, MFIBs were observed in both mother and the bud cells with similar frequency. In conditions in which MFIBs were not formed in cells, the straight elongated mitochondria as shown in Fig. 1B were not observed. This suggests that MFIBs contribute to the formation of elongated mitochondria.

MFIBs did not appear in log-phase cells, but rapidly began to appear early in the stationary phase (Fig. 1C). At the first stage of appearance of MFIBs, the fluorescence intensity was rather faint and a few inclusion bodies were seen in a cell. However, MFIBs gradually increased in fluorescence intensity in the cells that passed the log-phase. Early in the stationary phase, 5 distinct inclusion bodies, on average, appeared in a cell. At the stationary phase, autofluorescence persisted for 10∼20 sec under UV illumination.

### Isolation of mitochondria that retained MFIBs

**Fig. 2.**
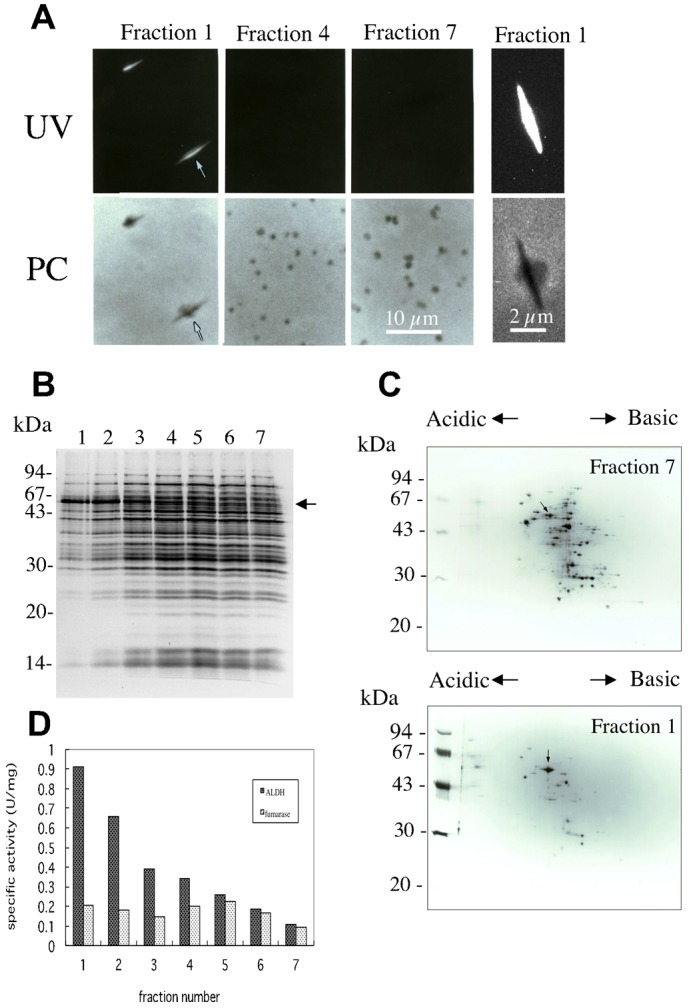
Fractionation of mitochondria that contain MFIBs. (A) Mitochondrial fractions that were obtained by differential centrifugations were fractionated once by discontinuous sucrose gradient centrifugation and then further fractionated by 30–60% sucrose gradient centrifugation. The band of mitochondria in a sucrose gradient was fractionated into 7 fractions of 1 ml each. Fraction 1 is the lowest part of the band and fraction 7 is the uppermost part. Left photographs represent fractions 1, 4, and 7, which were observed by phase contrast (PC) and fluorescence microscopy under UV excitation (UV). Arrows indicate mitochondria with MFIBs. Right photographs represent an enlarged mitochondrion that contains MFIBs. (B) Analysis of mitochondrial proteins. Protein components of each fraction collected from sucrose density gradient centrifugation were analyzed by SDS-PAGE. The position of 54-kDa protein is indicated by an arrow. (C) Two-dimensional gel electrophoresis of mitochondrial fraction. Mitochondrial fraction 7 that did not contain MFIBs and fraction 1 that contained MFIBs were loaded on the gels. The 54-kDa protein is indicated by an arrow. (D) Enzymatic activity of the mitochondrial fractions. Specific activities of fumarase and aldehyde dehydrogenase were determined for each mitochondrial fraction collected from a sucrose density gradient. Scale bars: 10 µm (2 µm, enlarged).

In order to isolate mitochondria that contained MFIBs, mitochondrial fractions that were obtained by differential centrifugation of homogenated spheroplasts were further fractionated by sucrose density gradient centrifugation. At first, mitochondrial fractions were loaded on a discontinuous gradient of 30%, 40%, 50%, and 60% sucrose. After centrifugation, mitochondria that retained MFIBs were recovered from the boundary between 40% and 50% sucrose. Next, the MFIB-rich mitochondrial fraction was loaded onto a system for 30–60% linear sucrose density gradient centrifugation. The band of mitochondria was recovered in 7 fractions and each fraction was observed by fluorescence microscopy (Fig. 2A). Small mitochondria that were obtained in the upper and middle fractions did not contain MFIBs (Fig. 2A, Fractions 4 and 7). On the other hand, large mitochondria that contained long MFIBs were recovered from lower fractions (Fig. 2A, Fraction 1). The shape and size of MFIBs in isolated mitochondria were very similar to those seen in living cells. The isolated mitochondria with MFIBs had an appearance like a balloon stuck with a needle (Fig. 2A). These mitochondria could be stained with DIOC_6_(3) (data not shown).

### Analyses of protein components of MFIBs

According to a prediction that MFIBs are composed of protein components, 7 fractions that were obtained from the sucrose gradient centrifugations were analyzed by SDS-PAGE (Fig. 2B). Each fraction had similar protein components. However, among them, only a 54-kDa protein was highly concentrated in fractions 1 and 2, the heavier fractions of mitochondria that contained large mitochondria with MFIBs. Therefore, we analyzed the protein components of fraction 1 by two-dimensional gel electrophoresis and compared them with those of fraction 7, which contained only small mitochondria (Fig. 2C). Among a number of mitochondrial proteins, only a protein of 54 kDa at the position of pI 6.0 was highly concentrated in fraction 1 (Fig. 2C, arrows). Subsequently, we determined the N-terminal amino acid sequence of the 54-kDa protein with a protein sequencer after transfer of the proteins on a PVDF membrane. The ten-amino-acid sequence was determined to be FSHLPMTVPI. A homology search on the Protein Information Resource (PIR) database indicated a unique and perfect match between this sequence and the sequence from the amino acids at positions 24 to 33 of mitochondrial aldehyde dehydrogenase Ald4p, which is encoded by *ALD4* (YOR374W).

To compare the enzyme activity among the mitochondrial fractions, the activities of aldehyde dehydrogenase were measured in 7 fractions, as was that of fumarase as a marker enzyme of mitochondria (Fig. 2D). The level of specific activity of fumarase was in the range of 0.1–0.2 U/mg protein in all mitochondrial fractions. On the other hand, as expected from the presence of aldehyde dehydrogenase Ald4p, specific activity of aldehyde dehydrogenase was distinctly high in the heavier mitochondrial fraction. Fraction 1 that contained MFIBs had 6-fold-higher activity than fraction 7 that did not contain MFIBs.

### Localization of Ald4p to MFIBs

**Fig. 3.**
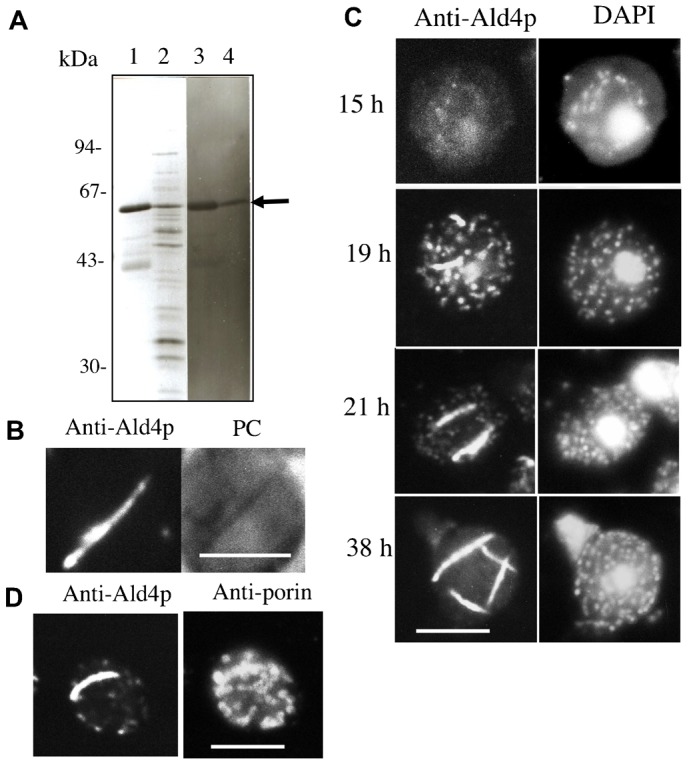
Immunoblotting and immunofluorescence microscopy with anti-Ald4p antibody. (A) Silver-staining and immunoblotting of commercially available aldehyde dehydrogenase from yeasts (lanes 1, 3) and mitochondrial fraction 1 (lanes 2, 4) with anti-Ald4p antibody. (B) Immunofluorescence microscopy of *S. cerevisiae* 3626 strain cells with anti-Ald4p antibody and the phase contrast image (PC). (C) Immunofluorescence microscopy of vegetative cells during growth with anti-Ald4p antibody. Strain 3626 was cultured in YPD medium as described in Fig. 1. Cells were fixed with 3.5% formaldehyde with time intervals, and processed for immunofluorescence microscopy and for DAPI staining. Immunofluorescence microscopy of cells was performed with anti-Ald4p antibody and a secondary antibody, Alexa 546 goat anti-rabbit IgG antibody. (D) Cells at 21-h culture were stained with anti-Ald4p antibody and Alexa 546 goat anti-rabbit IgG antibody to show MFIBs, and also stained with anti-porin antibody (Molecular Probe) and Alexa Fluor 488 goat anti-mouse IgG (Molecular Probe) to show the profile of mitochondria in a cell. Scale bars: 5 µm.

We raised a polyclonal antibody against commercially available aldehyde dehydrogenase (Ald4p) of *S. cerevisiae*. The antibody recognized only 54-kDa proteins among a number of proteins in mitochondrial fraction 1 as well as commercially available Ald4p by immunoblotting (Fig. 3A). Immunofluorescence microscopy of yeast cells with anti-Ald4p antibody clearly showed that anti-Ald4p antibody specifically binds to MFIBs (Fig. 3B).

In order to reveal the process of formation of MFIBs, immunofluorescence microscopy was performed with cells that were fixed at various stages during vegetative culture (Fig. 3C). MFIBs did not appear until 15 h from the onset of the culture, and the immunofluorescence by anti-Ald4p antibody was very faint in cells grown for 15 h. As the culture proceeded, immunofluorescence by anti-Ald4p antibody gradually became more intense, and Ald4p appeared as dots that corresponded to individual mitochondria, as revealed by DAPI staining of mitochondrial nucleoids during the transition from log-phase to stationary-phase cells at 19 h. Part of the Ald4p began to aggregate as short needle-like structures at 19 h. When cells reached the stationary phase, the needle-like structures with Ald4p became very thick. In contrast, the fluorescence intensity of dots determined by anti-Ald4p antibody gradually diminished from the rest of the mitochondria at 21-h and 38-h culture (Fig. 3C). Profiles of all mitochondria in a cell at stationary phase were stained by immunofluorescence microscopy using anti-porin antibody. In contrast, only a needle-like structure was intensely stained with anti-Ald4p antibody (Fig. 3D). These results demonstrated that Ald4p is aggregated to form MFIBs in an elongated mitochondrion during the transition from log-phase to stationary-phase.

In order to reveal the correlation of appearance of MFIBs and the expression level of Ald4p, mitochondria were isolated from cells that were cultured for 15 h, 21 h and 38 h. Those mitochondrial proteins were separated by SDS-PAGE and immunoblotting with anti-Ald4p antibody and anti-porin antibody was performed. As shown in supplementary material Fig. S1, the amount of Ald4p that was contained in the mitochondria from log-phase cells (15 h) was smaller than that in the mitochondria from early stationary-phase cells (21 h) and stationary-phase cells (38 h). These results suggested that the appearance of MFIBs well correlated with the expression level of Ald4p.

### MFIBs are not formed in Ald4p-deficient strains

**Fig. 4.**
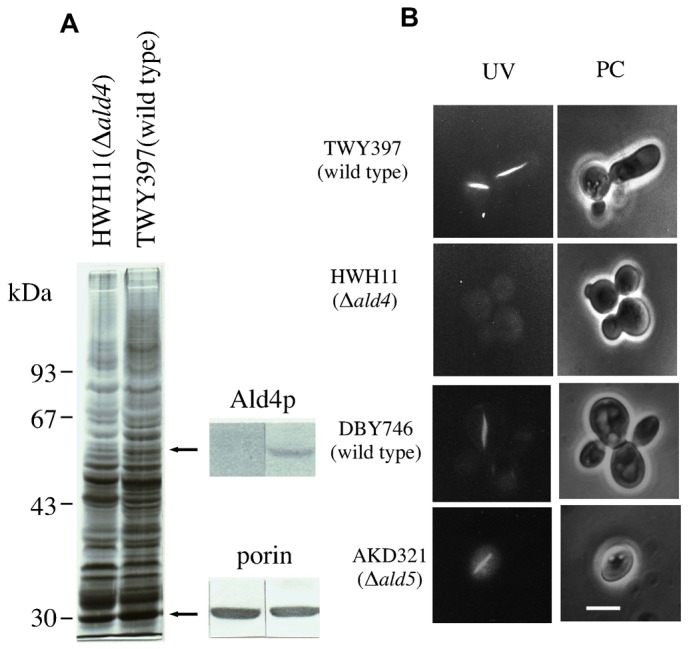
MFIB formation in Δ*ald4* cells and in Δ*ald5* cells. (A) SDS-PAGE and immunoblotting of mitochondria isolated from strains HWH11 (Δ*ald4*) and TWY397 (wild type). Mitochondria were isolated from both strains grown to stationary phase in YPE medium. Immunoblotting with anti-Ald4p antibody and anti-porin antibody was performed. (B) Observation of MFIBs in TWY397 strain (parent strain of Δ*ald4*), HWH11 (Δ*ald4*) strain, DBY746 strain (parent strain of Δ*ald5*), and AKD321 (Δ*ald5*) strain grown in YPE medium. Fluorescence images (UV) and the phase contrast images (PC) are shown. Scale bar: 5 µm.

There are two isozymes of mitochondrial aldehyde dehydrogenase in *S. cerevisiae*. Ald4p provides 80% of the activity, while the remaining 20% is due to Ald5p (Kurita and Nishida, 1999). Comparison and immunoblotting of mitochondrial fractions isolated from the strain HWH11 (Δ*ald4*) and the wild-type TWY397 strain confirmed that Ald4p was not detected in the Δ*ald4* strain (Fig. 4A). When the wild-type TWY397 and the strain HWH11 (Δ*ald4*) were cultured in YPE (containing 2% ethanol instead of 2% glucose) medium to the stationary phase, distinct MFIBs were observed in the parent strain TWY397, but were not observed in the Δ*ald4* strain (Fig. 4B). On the other hand, both the strain AKD321 (Δ*ald5*) and the parent strain DBY746 grown in YPE medium contained MFIBs early in the stationary phase. The percentage of cells that had MFIBs after 18-h culture of cells in YPE medium reached 70% in Δ*ald5* cells and the parent strain.

### Fine structure of MFIBs revealed by electron microscopy

**Fig. 5.**
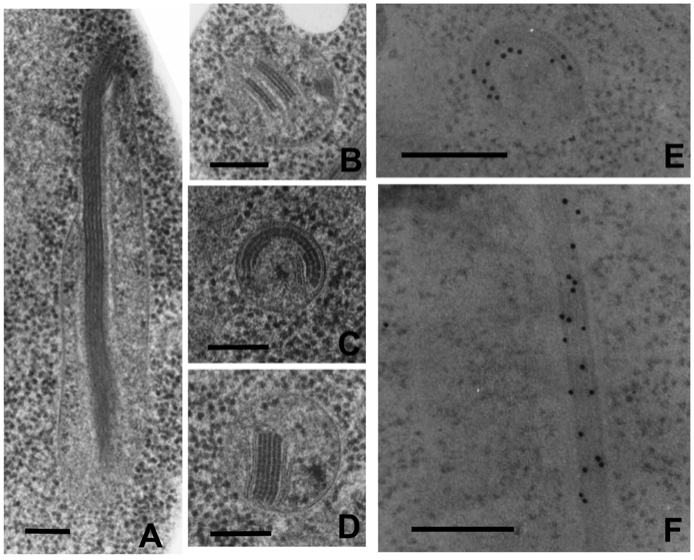
Freeze-substitution electron microscopy of intramitochondrial inclusion body. (A) A longitudinal section of inclusion body with five layers in elongated mitochondria. (B) A cross section of inclusion body with bilayer and triple layers between cristae. (C) A cross section of inclusion body with circular bilayer (partially triple layers). (D) A cross section of inclusion body with five layers sandwiched between cristae. (E) Immunoelectron microscopy of a cross section of inclusion body. (F) Immunoelectron microscopy of a longitudinal section of inclusion body. Scale bars: 200 nm.

To reveal the fine structure of MFIBs, cells of strain 3626 at stationary phase were fixed by rapid freezing followed by substitution with osmium acetone, and then processed for thin section electron microscopy. Sections were stained with both lead acetate and lead citrate. As a result, filamentous inclusion body with high electron density was distinctly observed in the matrix of elongated mitochondria. In a longitudinal section shown in Fig. 5A, this structure consisted of 5 layers of fine filaments with an average thickness of 10 nm, running parallel to each other along the long axis of a mitochondrion. In other longitudinal sections, the filamentous inclusion body consisted of 1–5 layers of fine filaments. The cross sections of the mitochondria with MFIBs are shown in Fig. 5B–D. There are several different types of stacking of the filamentous inclusion body. Fig. 5B shows that two and three layers of the fine filaments were separately sandwiched by a pair of cristae. Spherical layers of filaments were also seen (Fig. 5C). Fig. 5D shows 5 layers of fine filaments clearly sandwiched by two crista membranes. These microphotographs suggested the close spatial relationship of cristae and the filamentous inclusion body with high electron density. Immunoelectron microscopy with anti-Ald4p antibody showed that 10 nm gold-colloids specifically localized to the filamentous inclusion body in both cross and longitudinal sections (Fig. 5E,F). These results indicated that the filamentous inclusion body revealed by electron microscopy coincided with MFIBs revealed by fluorescence microscopy, in which Ald4p is contained as a major component.

### Occurrence of multiple inclusion bodies by overexpression of Ald4p

**Fig. 6.**
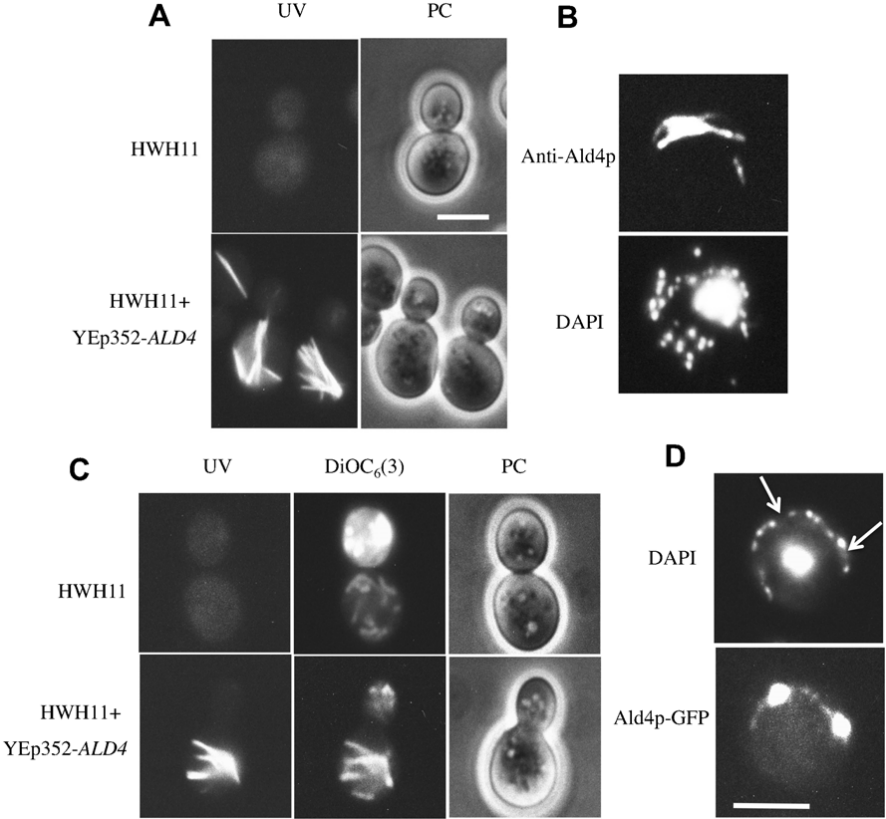
Formation of MFIBs by overexpression of *ALD4*. (A) Cells were cultured to the stationary phase in SD medium and observed for the presence of MFIBs under UV excitation. Fluorescence microscopy under UV excitation (UV) and the phase contrast images (PC) of HWH11 cells that lacked Ald4p, and HWH11 transformants in which the *ALD4* gene was expressed by YEp352-*ALD4* plasmid. (B) Immunofluorescence microscopy of HWH11 transformants with anti-Ald4p, and DAPI staining of the cell. (C) HWH11 cells and the transformants were vitally stained with DiOC_6_(3) and observed under UV excitation for MFIBs, under B excitation for mitochondria, and by phase contrast microscopy (PC). (D) Fluorescence microscopy of cells expressing Ald4p-GFP fusion protein. Ald4p-GFP fusion protein was expressed in HWH11 (Δ*ald4*) cells. Both DAPI staining image and the fluorescence image by GFP are shown. Scale bars: 5 µm.

The *ALD4* gene was subcloned into yeast/*E. coli* shuttle vector YEp352 and HWH11 (Δ*ald4*) strain was transformed by YEp352-*ALD4*. HWH11 strain and the transformant were cultured in SD medium supplemented with leucine and tryptophan to the stationary phase. As shown in Fig. 6A, a large number of MFIBs with strong autofluorescence were formed in the transformant in which Ald4p was overexpressed, whereas the parent Δ*ald4* cells did not form any MFIBs. We isolated mitochondria from both HWH11 (Δ*ald4*) strain and the transformant with YEp352-*ALD4* and confirmed by immunoblotting that Ald4p is expressed in the transformant (data not shown). In addition, we confirmed that MFIBs that are formed in the transformant contained Ald4p by immunofluorescence microscopy (Fig. 6B). HWH11 (Δ*ald4*) strain and the transformant were stained with DiOC_6_(3) to reveal the change of mitochondrial morphology. The HWH11 (Δ*ald4*) cells had short fragmented mitochondria without MFIBs (Fig. 6C). On the other hand, the transformant had many elongated mitochondria that overlapped with images of MFIBs.

If the Ald4p-GFP fusion protein also forms MFIBs, we can observe MFIBs without exposing the living cells to UV light. We constructed a fusion gene of *ALD4* and *GFP*, subcloned into YEp352 plasmid and expressed it in HWH11 (Δ*ald4*) cells that were cultured to stationary phase in a selectable synthetic medium. However, needle-like MFIBs that emitted green fluorescence were not observed, and instead, spherical aggregates appeared in those cells (Fig. 6D). Fluorescence of GFP showed a weak tubular structure and a few particles with very bright fluorescence along the cell periphery. Fluorescence of GFP overlapped with a string-of-beads appearance of the mt-nucleoids. These results indicated that Ald4p-GFP fusion protein formed spherical aggregates in particular regions in mitochondria. Aggregates of Ald4p-GFP were located in regions that were devoid of mt-nucleoids (Fig. 6D, arrows).

### Relationship between MFIBs and respiration competency

**Fig. 7.**
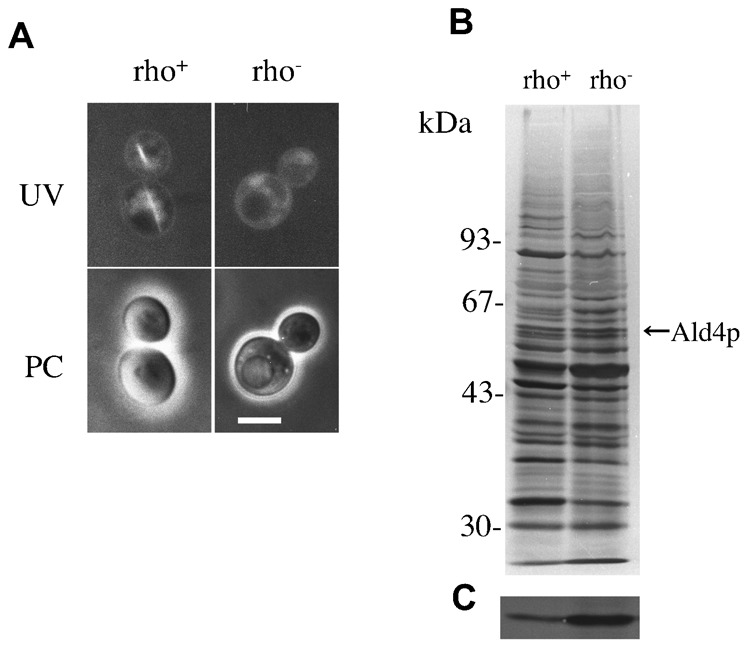
Relationship between MFIBs and respiration competency. (A) Inclusion body formation in wild-type cells (BY4741 rho^+^) and the respiration-deficient cells (BY4741 rho^−^). Fluorescence images under UV excitation (UV) and the phase contrast images (PC) are shown. (B) SDS-PAGE analysis of mitochondrial fractions from wild-type BY4741 cells (rho^+^) and the respiration-deficient cells (rho^−^). (C) Immunoblotting of panel B with anti-Ald4p antibody. Scale bar: 5 µm.

Although distinct MFIBs appeared in more than 50% of cells in BY4741 wild-type cells at the stationary phase, MFIBs were observed in less than 1% of the respiration-deficient (rho^−^) cells that were induced by the treatment of cells with ethidium bromide (supplementary material Table S1; Fig. 7A). To determine whether the quantity of Ald4p in mitochondria is affected by respiration deficiency, SDS-PAGE and immunoblotting of mitochondrial fractions obtained from both strains were performed. The results suggest that Ald4p is more abundant in mitochondria of respiration-deficient (rho^−^) cells than in mitochondria of the wild-type cells (Fig. 7B,C).

### Appearance of MFIBs in various yeasts

In the present study, we selected the 3626 strain from our laboratory stock strains as one that exhibited remarkable formation of MFIBs in YPD medium (Fig. 1). On the other hand, commonly used laboratory strain W303-1B also formed MFIBs at the stationary phase, as did laboratory strain BY4741 (Fig. 7). Long MFIBs that extended from end to end of the cells were not common in this strain. Instead, short MFIBs with weak fluorescence appeared in more than 50% of cells (supplementary material Fig. S2).

In order to investigate whether the occurrence of MFIBs is common in yeasts, 13 species among the genus *Saccharomyces* (Kurtzman and Fell, 1998) were cultured in YPD medium to stationary phase and tested for the appearance of MFIBs. As listed in supplementary material Table S2, except for two species, *S. kluyveri* and *S. kunashirensis*, 11 species formed MFIBs. However, the extent of the formation of MFIBs significantly varied, from less than 1% (*S. dairenensis*, *S. exiguus*) to more than 50% (*S. cerevisiae*, *S. servazzii*, *S. paradoxus*) among species. The species in which MFIBs emitted the strongest fluorescence was *S. paradoxus*. Six species, *Kluyveromyces lactis*, *Williopsis mrakii*, *Candida parapsilosis*, *Pichia jadinii*, *Trigonopsis variabilis*, and *Schizosaccharomyces pombe*, were cultured in both YPD medium and YPE (containing 2% ethanol) to the stationary phase. As a result, MFIBs were observed in two species, *Kluyveromyces lactis* and *Williopsis mrakii*, in both YPD and YPE media, but were not observed in the other 4 species in both media. Among species that formed MFIBs, 8 species, *S. servazzii*, *S. martiniae*, *S. paradoxus*, *S. spencerorum*, *S. unisporus*, *S. bayanus*, *K. lactis*, and *W. mrakii*, were tested for immunofluorescence by microscopy with anti-Ald4p antibody. As a result, MFIBs of all species tested were found to be stained with anti-Ald4p antibody of *S. cerevisiae* (supplementary material Fig. S3). These results indicated that the formation of MFIBs by Ald4p is not restricted to a particular strain of *S. cerevisiae*, but rather that MFIBs are a common intramitochondrial structure among the yeasts.

## DISCUSSION

### MFIBs are frequently observed in fluorescence microscopy of yeast cells

In our experiments, needle-like fluorescent structures in mitochondria (MFIBs) have been observed in several strains of *S. cerevisiae*, upon reaching the stationary phase. The frequency of occurrence of MFIBs significantly depends on the strains used. In a particular strain, MFIBs were observed in more than 80% of cells, but in other strains, these structures appeared in only 1% of cells. One reason for researchers missing these structures may be that their autofluorescence completely diminishes upon the chemical fixation (ethanol, glutaraldehyde, or formaldehyde fixation) of cells, which we routinely use for immunofluorescence microscopy and DAPI staining. In addition, there is often no reason for researchers to observe living cells under UV excitation. In fact, as shown in supplementary material Table S2, MFIBs are present in many species of yeast. However, so far, there has been little information on the components of these structures.

### Large mitochondria with MFIB can be separated from other mitochondrial populations

Among the yeast strains tested, we used the 3626 strain for the isolation of mitochondria containing MFIBs because this strain exhibited marked formation of MFIBs in cells that reached the stationary phase (Fig. 1). Using this strain, for the first time we successfully isolated mitochondria with MFIBs.

Large mitochondria with MFIBs are shaped like a balloon with a needle inside it (Fig. 2). This implies that needle-like MFIBs that emit autofluorescence are rather rigid structures, but do not penetrate mitochondrial membranes. We tried to isolate MFIBs from mitochondria after lysis of the mitochondrial membrane using a detergent such as 1% Nonidet P-40. However, this failed because solubilization of the mitochondrial membrane by detergent immediately resulted in collapse of the MFIBs (data not shown). Analysis of protein components of mitochondrial fractions by first- and second-dimensional gel electrophoresis clearly revealed that a 54-kDa Ald4p is abundant in MFIB-enriched mitochondria (Fig. 2).

Five isozymes that belong to the aldehyde dehydrogenase (ALDH) family have been found in the *S. cerevisiae* genome (Saccharomyces Genome Database). Three genes, *ALD2*, *ALD3*, and *ALD6*, among which *ALD6* encodes a major enzyme that functions in the cytoplasm, encode cytosolic ALDHs. On the other hand, mitochondrial ALDHs are encoded by two genes, *ALD4* and *ALD5*. The 54-kDa Ald4p is a major mitochondrial ALDH that is encoded by the *ALD4* gene; it provides more than 80% of the activity of mitochondrial ALDH (Wang et al., 1998; Kurita and Nishida, 1999). Although we cannot exclude the possibility of involvement of another mitochondrial ALDH, Ald5p, in the formation of MFIBs, we assume this is unlikely from the evidence that MFIBs did not appear in Ald4p-deficient cells, and that a lack of Ald5p has little influence on MFIB formation (Fig. 4B).

### Origin of autofluorescence emitted from MFIBs

The autofluorescence that was emitted from cells can be divided into at least two distinct spectral regions: one exciting at λmax 365 nm and emitting at λmax 445 nm (blue), and the other exciting at λmax 380 nm and λmax 440 nm and emitting at λmax 520 nm (green). The former autofluorescence is due to NADH and the latter is due to riboflavin, flavin coenzymes, and flavoproteins bound in the mitochondria (Aubin, 1979; Benson et al., 1979). Actually, our preliminary examination showed that the mitochondrial fraction that contained MFIBs exhibited fluorescence emission maxima at 450 nm, when excited at 365 nm (data not shown). As Ald4p binds NAD(P)^+^ or NAD(P)H as a coenzyme, autofluorescence emitted by MFIBs may be due to NAD(P)H in its bound form.

### Relationship among growth phase, metabolic state, and occurrence of MFIB

The production of mitochondrial K^+^-activated aldehyde dehydrogenase, Ald4p, is highly suppressed in the presence of glucose. Actually, mitochondrial ALDH activity is 1,000-fold higher in stationary-phase cells than in log-phase cells in culture using glucose as a carbon source (Jacobson and Bernofsky, 1974). This change in Ald4p activity well corresponds to the appearance of MFIBs during vegetative culture (Fig. 3; supplementary material Fig. S1). Almost all mitochondria in log-phase cells could not be stained by immunofluorescence microscopy with anti-Ald4p antibody (Fig. 3C). However, mitochondria in cells early in the stationary phase to the main part of the stationary phase were extensively stained with anti-Ald4p antibody. In the stationary phase, Ald4p largely aggregated to needle-like MFIBs and the fluorescence intensity of other parts of the mitochondria somewhat decreased (Fig. 3C). Yeast mitochondria dynamically fuse and divide during vegetative growth. It is possible that Ald4p, which was fully activated and accumulated in mitochondria, aggregated into paracrystalline inclusions in a particular region of the mitochondria for an unknown reason.

Ald4p plays a role in the oxidization of ethanol to acetate in mitochondria (Saigal et al., 1991; Tessier et al., 1998). Therefore, Ald4p production is activated after the diauxic phase of vegetative growth, in which energy production by glycolysis is changed to respiration by the oxidation of ethanol (Fig. 1C) (DeRisi et al., 1997). Actually, when *S. paradoxus* was cultured in YPD medium, YPG medium, or YPE medium, MFIBs began to appear in cells at 18-h culture, 6-h culture, and 3-h culture, respectively (data not shown). The appearance of MFIBs may be closely associated with the oxidation of ethanol. In addition, the expression of *ALD* genes depends on the strains used (Aranda and del Olmo, 2003). The difference in frequency of the occurrence of MFIBs and their size may be explained by the difference in expression level of *ALD4* among yeasts. The present study demonstrates the fact that Ald4p is not uniformly distributed in mitochondria, but this enzyme is accumulated or acts in a particular portion of mitochondria at a particular stage of vegetative growth. It remains to be determined whether polarized localization of other enzymes exists in a tubular network of mitochondria.

### Ultrastructure of MFIBs

Yotsuyanagi clearly demonstrated the presence of an intramitochondrial fibrous component (IMF) in *S. cerevisiae* and *Saccharomyces uvarum* by successful fixation using glutaraldehyde-osmium tetroxide (Yotsuyanagi, 1988). Although the chemical composition of IMF was not revealed, we assume from the structural similarity that MFIBs are identical to IMF. However, there are actually several differences between MFIBs and IMF. The width of continuous dark filament in MFIBs was 10 nm, but it was 20 nm in dark filament in IMF. This difference may be due to the difference of the fixation method of cells. MFIBs were formed in almost all respiration-competent cells of strain 3626, but the frequency of occurrence of MFIBs decreased to less than 1% in the respiration-deficient (rho^−^) cells. In contrast, IMF was found in respiration-deficient rho^0^ cells as well as respiration-competent cells. This may have been due to the difference of strains used. In respiration-competent cells, dark filaments of both MFIBs and IMF are commonly sandwiched between a pair of cristae, suggesting that the interaction of Ald4p and cristae is important for the assembly of Ald4p into MFIBs. The fact that, irrespective of sufficient accumulation of Ald4p, the formation of MFIBs is very rare in respiration-deficient cells, in which the development of cristae is poor, suggests the importance of interaction of Ald4p and cristae (Figs 5, 7).

It is noteworthy that careful electron microscopic observation showed that small oval mitochondria of up to 50/cell are predominant in stationary-phase cells, but one mitochondrion is significantly larger than the others in the cell (Stevens, 1981). This large mitochondrion might contain MFIBs. However, the majority of these electron microscopic studies used permanganate fixation, which would destroy the MFIBs. Therefore, its presence was missed during many observations of mitochondria (Yotsuyanagi, 1988). We expected that Ald4p-GFP fusion proteins would assemble into MFIBs, but this was not the case. In turn, Ald4p-GFP fusion proteins assembled into spherical structures in a specific region of the tubular mitochondria (Fig. 6D). It is likely that Ald4p interact with each other, but the GFP portion of the fusion protein hampers the formation of parallel arrays of Ald4p.

In conclusion, our results suggest that the metabolic changes from fermentation to ethanol oxidation, the increase of Ald4p concentration in mitochondria, and the interaction of Ald4p and mitochondrial cristae are the key determinants for the formation of MFIBs.

### Appearance of mitochondrial inclusion bodies in various organisms

The presence of intramitochondrial fibrous inclusion bodies or “crystalline inclusions” of 3 to 20 nm in thickness has been reported from electron microscopic observations of a number of cell types from various species during the past 50 years. In human liver cells (Wills, 1965), rat kidney cells (Suzuki and Mostofi, 1967), and rabbit thyroid gland (Nathaniel, 1980), fibrous inclusion bodies were localized to the matrix of mitochondria. Intramitochondrial fibrous inclusion bodies also occurred in pathological conditions. Human liver affected by chronic alcoholism had fibrous inclusions measured up to 3 µm in length in the mitochondrial matrix (Svoboda and Manning, 1964). On the other hand, rat liver cells that were injected with ethanol had fibrous inclusions in the matrix or intermembrane space between inner and outer mitochondrial membranes (Iseri et al., 1966). Crystal-like intramitochondrial inclusions were occasionally seen in both Parkinson's and Alzheimer's disease cybrid cells (Trimmer et al., 2000). However, the nature and function of the inclusion bodies had not been determined and detailed analysis of their chemical composition had not been performed.

The chemical composition of paracrystalline inclusions within mitochondria was first demonstrated in adult rat cardiomyocytes that were cultured in creatine-deficient medium (Eppenberger-Eberhardt et al., 1991). The paracrystalline inclusions appeared in the intermembrane spaces of outer and inner membranes of elongated large mitochondria. Immunofluorescence microscopy and immuno-gold labeling of freeze-substituted cardiomyocytes with anti-mitochondrial creatine kinase (Mi-CK) demonstrated that Mi-CK is a main component of mitochondrial inclusions (Eppenberger-Eberhardt et al., 1991). Mitochondrial myopathies are characterized by the presence of ragged-red (RR) fibers in muscle biopsy specimens. Characteristic aspects of the pathology of the RR fibers are the occurrence of abnormal mitochondria of highly ordered crystal-like inclusions (Stadhouders et al., 1994). Stadhouders et al. first demonstrated from immunoelectron microscopy that Mi-CK octamer is a major component of these mitochondrial inclusions (Stadhouders et al., 1994). Subsequently, the crystalline inclusions were isolated from creatine-depleted rat soleus muscle (O'Gorman et al., 1997). Mitochondrial creatine kinase is located on the outer side of the inner mitochondrial membrane.

We cannot conclude that Ald4p is the only component of MFIBs, even though it is clear that it is a main component of them. Purification of MFIBs will enable clarification of the complete range of components of MFIBs. The detailed analysis of filamentous dark structures of which MFIBs are composed will reveal how Ald4p assemble to form paracrystalline arrays. Our finding may lead to the development of genetic analyses for the mechanisms of MFIB formation.

## MATERIALS AND METHODS

### Strains and culture

Strains used in this study are listed in supplementary material Table S1. *Saccharomyces cerevisiae*, strain 3626, was used for the isolation of mitochondria that contain MFIBs. Cells were cultured aerobically at 30°C in YPD medium that contained 1% yeast extract (Oriental Yeast Co., Ltd., Tokyo, Japan), 2% peptone (Kyokuto Co., Ltd., Tokyo, Japan), and 2% glucose. YPG medium that contained 2% glycerol instead of glucose, YPE medium that contained 2% ethanol instead of glucose, or SD medium (0.67% yeast nitrogen base without amino acids, 2% glucose supplemented with amino acids and bases required) was also used. The rho^−^ strain was induced by treatment of BY4741 cells with 10 µg/ml ethidium bromide overnight. Cells after ethidium bromide treatment were plated on YPD and YPG plates, and cells that grew on YPD plates but not on YPG plates were selected. The maintenance of mtDNA in the rho^−^ cells was ascertained by DAPI staining. Other strains used for the observation of MFIBs are listed in supplementary material Table S2.

### Isolation of mitochondria that contained inclusion bodies

*S. cerevisiae*, 3626 strain, was cultured to the stationary phase in YPD medium. Cells were collected from 100 ml culture and were converted to spheroplasts by treatment with Zymolyase 20T (Seikagaku Kogyo Co., Ltd., Tokyo, Japan) according to a method described previously (Sato and Miyakawa, 2004). The spheroplasts were homogenized in 21.5 ml of 0.5 M sorbitol, 25 mM potassium phosphate buffer (pH 7.5) with Teflon Potter-Elvehjem homogenizer (930 rpm, 30 strokes), and then centrifuged at 1,600 × g for 5 min to sediment cell debris. The supernatant was loaded on a discontinuous gradient of 6 ml of 60% sucrose, 8 ml of 50% sucrose, 8 ml of 40% sucrose, and 8 ml of 30% sucrose containing 20 mM Tris-HCl (pH 7.5), 1 mM EDTA, 7 mM β-mercaptoethanol, and 0.4 mM PMSF, and then centrifuged at 80,000 × g for 1 h. The mitochondrial fractions that contained MFIBs were collected from the boundary between 50% and 40% sucrose. The mitochondrial fractions were carefully diluted twofold with distilled water and loaded onto a 60% sucrose–30% sucrose linear gradient and centrifuged at 80,000 × g for 1 h. Each 1 ml fraction was carefully fractionated from the centrifuge tubes and was observed by fluorescence microscopy to find MFIB-containing mitochondria.

### SDS-PAGE and immunoblotting

For SDS-PAGE, an aliquot of each sample (15 µl) was mixed with 5 µl of 4× SDS sample buffer and loaded on a 15% polyacrylamide gel as described previously (Laemmli, 1970). Proteins on gels were detected by the silver-staining method (Oakley et al., 1980). The molecular-mass markers (Pharmacia, Uppsala, Sweden) used were phosphorylase b (94 kDa), bovine serum albumin (67 kDa), ovalbumin (43 kDa), carbonic anhydrase (30 kDa), soybean trypsin inhibitor (20 kDa) and α-lactalbumin (14.4 kDa).

The method used for immunoblotting was based on a method described previously (Towbin et al., 1979). Antibodies that reacted with the antigens on polyvinylidene difluoride filters were detected by the immunoperoxidase procedure using the Vectastain ABC kit (Vector Laboratories Inc., Burlingame, CA, USA) as indicated in the instruction manual.

For preparation of polyclonal antibody against Ald4p, commercially available aldehyde dehydrogenase from the yeast *S. cerevisiae* (E.C. 1.2.1.5, Sigma) was used as an antigen against rabbits. As commercially available aldehyde dehydrogenase was not homogeneous, we used a band of 54 kDa excised from SDS-polyacrylamide gels as an antigen protein. Antisera were tested by immunoblotting of strips of the PVDF membranes. The antibody was affinity purified with Ald4p that was transferred to PVDF membranes and used at 1:1,000 dilution for immunoblotting.

### Two-dimensional (2D) gel electrophoresis

2D gel electrophoresis was performed according to a method described previously (Oh-Ishi et al., 1998). Isolated mitochondria were suspended in sample buffer (5 M urea, 1 M thiourea, 20 mM β-mercaptoethanol, 2% Nonidet P-40) and sonicated on ice. After centrifugation at 27,000 g for 30 min, the supernatants were applied on the acidic side of isoelectric focusing agarose gel for the first-dimension electrophoresis. The second-dimension SDS-PAGE was performed on 10% polyacrylamide gel by a method described previously (Laemmli, 1970). The gels after the second-dimension electrophoresis were silver-stained or transferred to PVDF membranes.

### Analysis of amino acid sequence

Proteins from mitochondria were separated by 2-D gel electrophoresis and transferred to a PVDF membrane. The membrane was stained with amido black and each spot was excised. The N-terminal amino acid sequence was determined with a Shimadzu Protein sequencer, PPSQ-21.

### Enzyme assays

Aldehyde dehydrogenase activity was assayed by measuring the change of absorption at a wavelength of 340 nm due to the production of NADH in a reaction mixture of 100 mM KCl, 10 mM DTT, 5 mM EDTA, 1 mM PMSF, 2 mM propionaldehyde, and 0.5 mM NAD^+^. Fumarase activity was assayed by measuring the change of absorption at a wavelength of 250 nm in a reaction mixture of 50 mM L-malic acid and 90 mM potassium phosphate buffer, pH 7.4 (Kanarek and Hill, 1964).

### Immunofluorescence microscopy

Cells were fixed with 3.5% formaldehyde in the culture medium for 1 h at room temperature. After washing with SP buffer (0.8 M sorbitol, 25 mM potassium phosphate buffer, pH 7.5), cells were pretreated with 0.4 M β-mercaptoethanol for 30 min. After washing with SP buffer, cells were treated with Zymolyase 20T in SP buffer for 30 min to digest the cell wall. Immunofluorescence microscopy was performed according to a method described previously (Kilmartin and Adams, 1984). Primary antibodies used were anti-Ald4p antibody (rabbit polyclonal, 1:50 dilution, this study) and anti-porin (mouse, monoclonal, Molecular Probe Inc., 1:20 dilution). Alexa 546 goat anti-rabbit IgG (Molecular Probe) and Alexa Fluor 488 goat anti-mouse IgG (Molecular Probe) were used as secondary antibodies at 1:500 dilution. Immunostained cells were double-stained with DAPI (1 µg/ml). Preimmune serum was used in the same method as a control.

### Freeze-substitution electron microscopy

For freeze substitution, stationary-phase cells of *S. cerevisiae* strain 3626 grown on YPD medium were sandwiched between two copper grids, each carrying a thin layer of cells, and were quickly plunged into propane cooled with liquid nitrogen with Reicher-Jung KF-80. The procedures for substitution in 2% OsO_4_-acetone at −79°C and in anhydrous acetone at increasing temperatures and embedding by Epon-Araldite resin were as described previously (Tanaka and Kanbe, 1986). Serial thin sections cut to 80 nm were made using a Reichert Ultracut OmU4 ultramicrotome with a diamond knife, and were collected on Formvar-coated single-slot grids. They were stained with uranyl acetate and lead citrate, and viewed with a JEOL 100 SX electron microscope operated at 80 kV.

For immunogold labeling, cells were frozen rapidly in propane cooled with liquid nitrogen. Substitution was carried out with acetone at −79°C and in anhydrous acetone at increasing temperatures, and embedding by Epon-Araldite resin was carried out as described above. The sections were immunostained with anti-Ald4p antibodies at a dilution of 1:100, and then with 10-nm gold particle-conjugated goat anti-rabbit IgG (British BioCell International, Cardiff, UK). They were double-stained with uranyl acetate and lead citrate, and viewed with a Philips CM 120 electron microscope operated at 100 kV.

### Transformation

The *ALD4* gene, including 560 bp upstream and 530 bp downstream, was amplified by PCR using forward primer 5′-TGCATTACCGGCAGTTGCTC-3′ and reverse primer 5′-GCTGCTAACTTGGGTGGTGT-3′. The amplified gene was inserted into pGEM-T Easy Vector (Promega) by TA cloning according to the instruction manual. The cloned *ALD4* gene was digested by *Eco*R I and subcloned into yeast/*E. coli* shuttle vector YEp352. HWH11 strain was transformed by YEp352-*ALD4* by lithium acetate methods and *URA3* transformants were selected. In this report, we designate the *ALD2* gene as the *ALD4* gene according to the Yeast Protein Database (Wang et al., 1998).

### Construction of Ald4p-GFP fusion protein

The *ALD4* gene, including 560 bp upstream and excluding the termination codon, was amplified by PCR with forward primer 5′-TGCATTACCGGCAGTTGCTC-3′ and reverse primer 5′-ggggtaccccCTCGTCCAATTTGGCACGGA-3′. Small capitals show the nucleotides of the *Kpn* I site. The cloned gene was inserted into pGEM-T Easy Vector (Promega) by TA cloning. The *GFP* gene, including 21 bp upstream and 33 bp downstream, was amplified from pGFPuv vector by PCR with forward primer 5′-CCCCGGGTACCGGTAGAAAA-3′ and reverse primer 5′-TAATGGTAGCGACCGGCGCT-3′. The cloned gene was inserted into pGEM-T Vector (Promega) by TA cloning. The cloned *ALD4* gene and the *GFP* gene were digested with *Eco*RI and *Kpn* I and both DNA fragments were ligated and inserted into the *Eco*R I-*Kpn* I site of YEp352.

## Supplementary Material

Supplementary Material
