# Supplementary material for: Aldehyde dehydrogenase, Ald4p, is a major component of mitochondrial fluorescent inclusion bodies in the yeast Saccharomyces cerevisiae
Source: Biol Open. 2014 Apr 25;3(5):387–96. doi: 10.1242/bio.20147138 (PMC4021361; doi:10.1242/bio.20147138)
Supplement: Supplementary Material [file supp_3_5_387__index.html]

Aldehyde dehydrogenase, Ald4p, is a major component of mitochondrial fluorescent inclusion bodies in the yeast Saccharomyces cerevisiae — Supplementary Material 

# Aldehyde dehydrogenase, Ald4p, is a major component of mitochondrial fluorescent inclusion bodies in the yeast *Saccharomyces cerevisiae*

## bio.20147138 Supplementary Material

**Files in this Data Supplement:**

- Supplementary Material - Yoshiko Misonou et al. doi: 10.1242/bio.20147138
