# Supplementary material for: Aldehyde dehydrogenase, Ald4p, is a major component of mitochondrial fluorescent inclusion bodies in the yeast Saccharomyces cerevisiae
Source: Biol Open. 2014 Apr 25;3(5):387–96. doi: 10.1242/bio.20147138 (PMC4021361; doi:10.1242/bio.20147138)
Supplement: Supplementary Material [file supp_bio.20147138_bio.20147138-s1.pdf]

Biology Open (2014) 000, 1–10 doi:10.1242/bio.20147138

Supplementary Material

Yoshiko Misonou et al. doi: 10.1242/bio.20147138

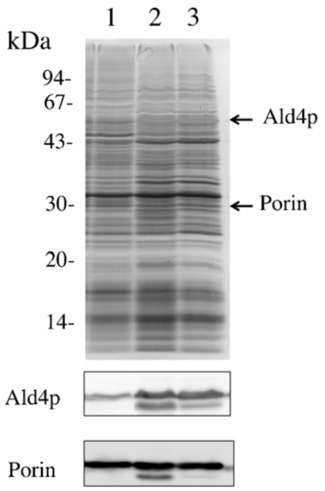

Fig. S1. Immunoblotting of mitochondria that were isolated from cells at various culture stages. *S. cerevisiae* strain 3626 cells were cultured in YPD medium as in Fig. 3 and collected at 15 h, 21 h and 38 h. Mitochondria were isolated from those cells by differential centrifugations and a discontinuous sucrose gradient centrifugation. The same amount of mitochondrial protein (14 µg/lane) from 15-h grown cells (lane 1), 21-h grown cells (lane 2) and 38-h grown cells (lane 3) was separated by SDS-PAGE and immunoblotting with anti-Ald4p antibody and anti-porin antibody was performed.

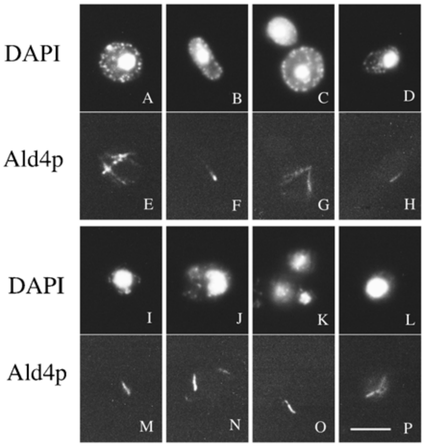

Fig. S3. DAPI staining and immunofluorescence microscopy of various yeasts. Stationary-phase cells from various yeast species were observed by immunofluorescence microscopy with anti-Ald4p antibody and by DAPI staining. *Saccharomyces servazzii* (A,E); *Saccharomyces martiniae* (B,F); *Saccharomyces paradoxus* (C,G); *Saccharomyces spencerorum* (D,H); *Saccharomyces unisporus* (I,M); *Saccharomyces bayanus* (J,N); *Kluyveromyces lactis* (K,O); *Williopsis mrakii* (L,P). Panels A–D,I–L are DAPI-staining images, panels E–H,M–P are immunofluorescence images. Scale bar: 5 µm.

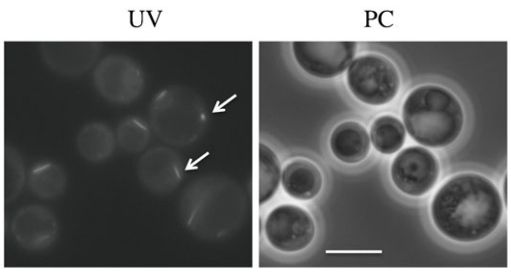

Fig. S2. MFIB formation in a commonly used laboratory strain. A laboratory strain, W303-1B, was cultured to stationary phase in YPD medium and observed under UV excitation and by phase contrast microscopy (PC). Arrows indicate short fragments of MFIBs. Scale bar: 5 µm.

Biology Open

Biology Open (2014) 000, 1–10 doi:10.1242/bio.20147138

Table S1. *S. cerevisiae* strains used in this study

Strain	Genotype	Source
3626	*MATα*	This study
TWY397	*MATα ura3 his7 trp1 leu2*	Wang et al., 1998
HWH11	*MATα ura3 his7 trp1 leu2 Δald2::HIS7*	Wang et al., 1998
DBY746	*MATα his3 leu2-3 leu2-112 trp1-289 ura3-52*	Kurita and Nishida, 1999
AKD321	*MATα his3 leu2-3 leu2-112 trp1-289 ura3-52 Δald5::LEU2*	Kurita and Nishida, 1999
W303-1B	*MATa ade2-1 can1-100 ura3-1 leu2-3,112 trp1-1 his3-11,15*	Thomas and Rothstein, 1989
BY4741	*MATa his3Δ1 leu2Δ0 met15Δ0 ura3Δ0*	Baker Brachmann et al., 1998

ALD2 is synonym to *ALD4*.

Table S2. Appearance of mitochondrial fluorescent inclusion bodies (MFIBs) in various yeasts

Species	Strains	MFIBs (%)	Anti-Ald4p
Saccharomyces cerevisiae	3626	+	+
Saccharomyces cerevisiae	BY4741 rho+	+	+
Saccharomyces cerevisiae	BY4741 rho−	±	+
Saccharomyces cerevisiae	W303-1B	+	n.d.
[1]*Saccharomyces dairenensis*	NBRC 0211	±	n.d.
[2]*Saccharomyces kluyveri*	NBRC 1685	−	n.d.
[3]*Saccharomyces servazzii*	NBRC 1838	+	+
[4]*Saccharomyces unisporus*	NBRC 0316	+	+
[5]*Saccharomyces exiguus*	NBRC 1128	±	n.d.
Saccharomyces bayanus	NBRC 11022	+	+
[6]*Saccharomyces castellii*	NBRC 1992	+	n.d.
Saccharomyces paradoxus	NBRC 10609	+	+
Saccharomyces pastorianus	NBRC 11024	+	n.d.
[7]*Saccharomyces spencerorum*	NBRC 10851	+	+
[8]*Saccharomyces kunashirensis*	NBRC 10915	−	n.d.
[9]*Saccharomyces martiniae*	NBRC 0752	+	+
Kluyveromyces lactis	2359/152	+	+
[10]*Williopsis mrakii*	CBS 1707	+	+
Candida parapsilosis	SR 23	−	n.d.
[11]*Pichia jadinii*	CBS 1600	−	n.d.
Trigonopsis variabilis	HUT 7537	−	n.d.
Schizosaccharomyces pombe	NCYC 132	−	n.d.

MFIBs (%) is expressed as the percentage of cells that retained inclusion body; +: >50%, ±: <1%, −: 0%. All strains were cultured to the stationary phase in YPD medium for 30–40 h and the presence of inclusion body was examined by fluorescence microscopy. Immunofluorescence microscopy of various strains with anti-Ald4p antibody was performed as described in Materials and Methods. Irrespective of the level of appearance, strains in which needle-like structures were stained with anti-Aldp4 antibody were expressed as +. Classification of species was based on Kurtzman and Fell, 1998. Present classification by Kurtzman et al., 2011 is as follows: [1]*Naumovozyma dairenensis*, [2]*Lachancea kluyveri*, [3]*Kazachstania servazzii*, [4]*Kazachstania unisporus*, [5]*Kazachstania exiguus*, [6]*Naumovozyma castellii*, [7]*Kazachstania spencerorum*, [8]*Kazachstania kunashirensis*, [9]*Kazachstania martiniae*, [10]*Lindnera mrakii*, [11]*Lindnera jadinii*.
